# *N*-[(*E*)-3,4-Dimeth­oxy­benzyl­idene]-2,3-dimethyl­aniline

**DOI:** 10.1107/S1600536811032892

**Published:** 2011-08-27

**Authors:** M. Nawaz Tahir, Muhammad Ilyas Tariq, Riaz H. Tariq

**Affiliations:** a Department of Physics, University of Sargodha, Sargodha, Pakistan; b Department of Chemistry, University of Sargodha, Sargodha, Pakistan; c Institute of Chemical and Pharmaceutical Sciences, The University of Faisalabad, Faisalabad, Pakistan

## Abstract

In the title compound, C_17_H_19_NO_2_, the aromatic rings are oriented at a dihedral angle of 59.27 (12)°. In the crystal, inversion dimers linked by pairs of weak C—H⋯O inter­actions generate *R*_2_^2^(12) loops.

## Related literature

For related structures, see: Sarfraz *et al.* (2010 ▶); Tahir *et al.* (2010**a* ▶,b* ▶); Tariq *et al.* (2010 ▶).
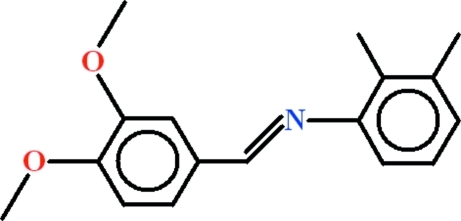


## Experimental

### 

#### Crystal data

C_17_H_19_NO_2_
*M* *_r_* = 269.33
Triclinic, 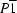

*a* = 7.0144 (4) Å
*b* = 7.4488 (4) Å
*c* = 15.6202 (8) Å
α = 81.987 (2)°
β = 81.009 (3)°
γ = 73.527 (2)°
*V* = 769.12 (7) Å^3^
*Z* = 2
Mo *K*α radiation
μ = 0.08 mm^−1^
*T* = 296 K
0.34 × 0.25 × 0.22 mm

#### Data collection

Bruker Kappa APEXII CCD diffractometer
Absorption correction: multi-scan (*SADABS*; Bruker, 2005 ▶) *T*_min_ = 0.972, *T*_max_ = 0.983
8993 measured reflections
3688 independent reflections
2028 reflections with *I* > 2σ(*I*)
*R*_int_ = 0.019

#### Refinement

*R*[*F* ^2^ > 2σ(*F* ^2^)] = 0.061
*wR*(*F* ^2^) = 0.211
*S* = 1.08
3688 reflections
185 parameters
H-atom parameters constrained
Δρ_max_ = 0.19 e Å^−3^
Δρ_min_ = −0.18 e Å^−3^

### 

Data collection: *APEX2* (Bruker, 2009 ▶); cell refinement: *SAINT* (Bruker, 2009 ▶); data reduction: *SAINT*; program(s) used to solve structure: *SHELXS97* (Sheldrick, 2008 ▶); program(s) used to refine structure: *SHELXL97* (Sheldrick, 2008 ▶); molecular graphics: *ORTEP-3 for Windows* (Farrugia, 1997 ▶) and *PLATON* (Spek, 2009 ▶); software used to prepare material for publication: *WinGX* (Farrugia, 1999 ▶) and *PLATON*.

## Supplementary Material

Crystal structure: contains datablock(s) global, I. DOI: 10.1107/S1600536811032892/hb6358sup1.cif

Structure factors: contains datablock(s) I. DOI: 10.1107/S1600536811032892/hb6358Isup2.hkl

Supplementary material file. DOI: 10.1107/S1600536811032892/hb6358Isup3.cml

Additional supplementary materials:  crystallographic information; 3D view; checkCIF report

## Figures and Tables

**Table 1:** Hydrogen-bond geometry (Å, °)

*D*—H⋯*A*	*D*—H	H⋯*A*	*D*⋯*A*	*D*—H⋯*A*
C16—H16*B*⋯O2^i^	0.96	2.51	3.454 (3)	167

Symmetry code: (i) 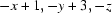.

**Fig. 1.**
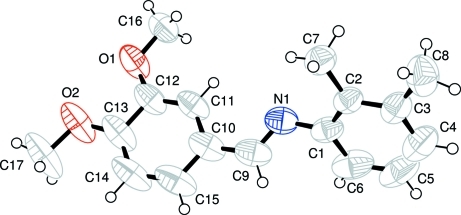
View of (I) with displacement ellipsoids drawn at the 50% probability level.

**Fig. 2.**
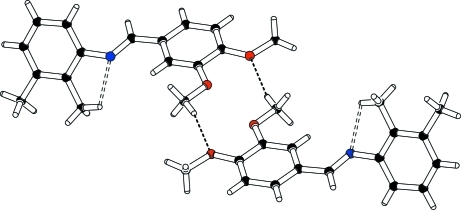
The partial packing of (I) showing that molecules form inversion dimers. The dotted lines represent the H-bondings.

## supplementary crystallographic information

### Comment

We have reported crystal structures of Schiff bases containining 2,3-dimethylaniline moiety *i.e.* (II) *N*-[(*E*)-4-chlorobenzylidene] -2,3-dimethylaniline (Tahir *et al.*, 2010*a*), (III) *i.e.*, 2-[(*E*)-(2,3-dimethylphenyl)iminomethyl]phenol (Tahir *et al.*, 2010*b*), (IV) *i.e.*, *N*-{(*E*)-[4-(dimethylamino)phenyl] methylidene}-2,3-dimethylaniline (Sarfraz *et al.*, 2010) and (V) *i.e.*, (2*Z*)-2-[(2,3-dimethylphenyl)imino]-1,2-diphenylethanone (Tariq *et al.*, 2010). The title compound (I), (Fig. 1) is in continuation to synthesize various compounds of 2,3-dimethylaniline.

In (I), the group A (C1—C8/N1) of 2,3-dimethylaniline and the group B (C9—C17/O1/O2) of 3,4-dimethoxybenzaldehyde are close to planar with r.m.s. deviations of 0.010 and 0.041 Å, respectively. The dihedral angle between A/B is 60.57 (4)°. The molecules are linked in the form of dimers due to inter-molecular hydrogen bonds of C—H···O type with *R*_2_^2^(12) ring motif (Table 1, Fig. 2). There does not exist any kind of significant π-interactions.

### Experimental

Equimolar quantities of 2,3-dimethylaniline and 3,4-dimethoxybenzaldehyde were refluxed in methanol for 30 min. The solution was kept at room temperature which affoarded colorless prisms after 48 h.

### Refinement

The H-atoms were positioned geometrically (C–H = 0.93–0.96 Å) and refined as riding with *U*_iso_(H) = *xU*_eq_(C), where *x* = 1.5 for methyl and *x* = 1.2 for other H-atoms.

### Crystal data

**Table:**

C_17_H_19_NO_2_	*Z* = 2
*M**_r_* = 269.33	*F*(000) = 288
Triclinic, *P*1	*D*_x_ = 1.163 Mg m^−^^3^
Hall symbol: -P 1	Mo *K*α radiation, λ = 0.71073 Å
*a* = 7.0144 (4) Å	Cell parameters from 2028 reflections
*b* = 7.4488 (4) Å	θ = 2.7–28.4°
*c* = 15.6202 (8) Å	µ = 0.08 mm^−^^1^
α = 81.987 (2)°	*T* = 296 K
β = 81.009 (3)°	Prism, colorless
γ = 73.527 (2)°	0.34 × 0.25 × 0.22 mm
*V* = 769.12 (7) Å^3^	

### Data collection

**Table:**

Bruker Kappa APEXII CCD diffractometer	3688 independent reflections
Radiation source: fine-focus sealed tube	2028 reflections with *I* > 2σ(*I*)
graphite	*R*_int_ = 0.019
Detector resolution: 7.50 pixels mm^-1^	θ_max_ = 28.4°, θ_min_ = 2.7°
ω scans	*h* = −9→8
Absorption correction: multi-scan (*SADABS*; Bruker, 2005)	*k* = −9→9
*T*_min_ = 0.972, *T*_max_ = 0.983	*l* = −20→20
8993 measured reflections	

### Refinement

**Table:**

Refinement on *F*^2^	Primary atom site location: structure-invariant direct methods
Least-squares matrix: full	Secondary atom site location: difference Fourier map
*R*[*F*^2^ > 2σ(*F*^2^)] = 0.061	Hydrogen site location: inferred from neighbouring sites
*wR*(*F*^2^) = 0.211	H-atom parameters constrained
*S* = 1.08	*w* = 1/[σ^2^(*F*_o_^2^) + (0.0973*P*)^2^ + 0.0736*P*] where *P* = (*F*_o_^2^ + 2*F*_c_^2^)/3
3688 reflections	(Δ/σ)_max_ < 0.001
185 parameters	Δρ_max_ = 0.19 e Å^−^^3^
0 restraints	Δρ_min_ = −0.18 e Å^−^^3^

### Special details

**Table:**

Geometry. Bond distances, angles *etc*. have been calculated using the rounded fractional coordinates. All su's are estimated from the variances of the (full) variance-covariance matrix. The cell e.s.d.'s are taken into account in the estimation of distances, angles and torsion angles
Refinement. Refinement of *F*^2^ against ALL reflections. The weighted *R*-factor *wR* and goodness of fit *S* are based on *F*^2^, conventional *R*-factors *R* are based on *F*, with *F* set to zero for negative *F*^2^. The threshold expression of *F*^2^ > σ(*F*^2^) is used only for calculating *R*-factors(gt) *etc*. and is not relevant to the choice of reflections for refinement. *R*-factors based on *F*^2^ are statistically about twice as large as those based on *F*, and *R*- factors based on ALL data will be even larger.

### Fractional atomic coordinates and isotropic or equivalent isotropic displacement parameters (Å^2^)

**Table:**

	*x*	*y*	*z*	*U*_iso_*/*U*_eq_	
O1	0.4243 (2)	1.3278 (2)	0.06049 (11)	0.1032 (6)	
O2	0.0678 (3)	1.5334 (3)	0.09093 (12)	0.1237 (7)	
N1	0.4479 (2)	0.7156 (3)	0.26807 (10)	0.0781 (6)	
C1	0.4907 (3)	0.5385 (3)	0.31797 (12)	0.0724 (7)	
C2	0.6399 (3)	0.4985 (2)	0.37272 (11)	0.0653 (6)	
C3	0.6892 (3)	0.3227 (3)	0.42177 (13)	0.0801 (7)	
C4	0.5942 (5)	0.1908 (3)	0.41376 (19)	0.1067 (10)	
C5	0.4515 (5)	0.2251 (4)	0.3599 (2)	0.1191 (11)	
C6	0.3959 (4)	0.4001 (4)	0.31104 (18)	0.1058 (10)	
C7	0.7449 (3)	0.6439 (3)	0.38007 (15)	0.0814 (7)	
C8	0.8467 (4)	0.2768 (3)	0.48259 (17)	0.1107 (10)	
C9	0.2673 (3)	0.8109 (4)	0.26475 (13)	0.0873 (8)	
C10	0.2098 (3)	0.9939 (4)	0.21549 (13)	0.0872 (8)	
C11	0.3509 (3)	1.0665 (3)	0.15843 (12)	0.0804 (7)	
C12	0.2991 (3)	1.2443 (3)	0.11668 (13)	0.0845 (7)	
C13	0.1014 (3)	1.3574 (4)	0.13272 (14)	0.0982 (9)	
C14	−0.0383 (3)	1.2866 (5)	0.18777 (16)	0.1098 (12)	
C15	0.0153 (3)	1.1059 (5)	0.22878 (16)	0.1091 (12)	
C16	0.6230 (3)	1.2208 (3)	0.03706 (18)	0.1017 (9)	
C17	−0.1297 (4)	1.6586 (5)	0.1040 (2)	0.1511 (14)	
H4	0.62832	0.07416	0.44610	0.1282*	
H5	0.38993	0.13205	0.35542	0.1429*	
H6	0.29718	0.42404	0.27438	0.1267*	
H7A	0.71156	0.74586	0.33535	0.1221*	
H7B	0.88706	0.58857	0.37339	0.1221*	
H7C	0.70333	0.69060	0.43618	0.1221*	
H8A	0.84583	0.15970	0.51705	0.1659*	
H8B	0.81876	0.37504	0.52020	0.1659*	
H8C	0.97589	0.26637	0.44934	0.1659*	
H9	0.16648	0.76071	0.29555	0.1047*	
H11	0.48208	0.99265	0.14874	0.0964*	
H14	−0.16955	1.36030	0.19753	0.1317*	
H15	−0.08069	1.05903	0.26580	0.1308*	
H16A	0.69124	1.18297	0.08799	0.1523*	
H16B	0.69194	1.29576	−0.00456	0.1523*	
H16C	0.62060	1.11121	0.01184	0.1523*	
H17A	−0.22229	1.60982	0.08122	0.2265*	
H17B	−0.12956	1.78039	0.07430	0.2265*	
H17C	−0.16937	1.66912	0.16514	0.2265*	

### Atomic displacement parameters (Å^2^)

**Table:**

	*U*^11^	*U*^22^	*U*^33^	*U*^12^	*U*^13^	*U*^23^
O1	0.0793 (9)	0.1041 (11)	0.0950 (11)	0.0173 (8)	−0.0053 (8)	0.0011 (8)
O2	0.0955 (11)	0.1343 (14)	0.0975 (12)	0.0434 (10)	−0.0131 (9)	−0.0186 (10)
N1	0.0689 (10)	0.1016 (12)	0.0657 (10)	−0.0269 (9)	−0.0016 (7)	−0.0135 (8)
C1	0.0726 (11)	0.0870 (13)	0.0648 (10)	−0.0362 (10)	0.0107 (9)	−0.0220 (9)
C2	0.0683 (10)	0.0677 (11)	0.0609 (10)	−0.0251 (8)	0.0103 (8)	−0.0157 (8)
C3	0.0919 (13)	0.0699 (12)	0.0725 (12)	−0.0256 (10)	0.0236 (10)	−0.0164 (9)
C4	0.133 (2)	0.0838 (15)	0.1044 (19)	−0.0482 (15)	0.0309 (16)	−0.0238 (13)
C5	0.144 (2)	0.1027 (19)	0.133 (2)	−0.0808 (18)	0.034 (2)	−0.0442 (18)
C6	0.1058 (17)	0.139 (2)	0.0977 (17)	−0.0668 (16)	0.0115 (13)	−0.0523 (16)
C7	0.0819 (12)	0.0803 (12)	0.0893 (14)	−0.0328 (10)	−0.0139 (10)	−0.0060 (10)
C8	0.124 (2)	0.0967 (16)	0.0917 (17)	−0.0090 (15)	−0.0065 (15)	0.0061 (13)
C9	0.0686 (12)	0.1318 (18)	0.0640 (12)	−0.0304 (12)	−0.0026 (9)	−0.0174 (12)
C10	0.0633 (11)	0.1337 (19)	0.0587 (11)	−0.0106 (11)	−0.0090 (9)	−0.0217 (12)
C11	0.0590 (10)	0.1120 (16)	0.0585 (10)	0.0010 (10)	−0.0108 (8)	−0.0156 (10)
C12	0.0670 (11)	0.1122 (16)	0.0570 (11)	0.0084 (11)	−0.0100 (9)	−0.0159 (10)
C13	0.0765 (13)	0.130 (2)	0.0626 (12)	0.0260 (13)	−0.0180 (10)	−0.0259 (12)
C14	0.0621 (12)	0.167 (3)	0.0755 (14)	0.0215 (14)	−0.0105 (11)	−0.0365 (15)
C15	0.0625 (12)	0.178 (3)	0.0730 (14)	−0.0054 (15)	−0.0046 (10)	−0.0264 (16)
C16	0.0696 (12)	0.0984 (16)	0.122 (2)	−0.0017 (11)	−0.0060 (12)	−0.0103 (14)
C17	0.114 (2)	0.163 (3)	0.118 (2)	0.0679 (19)	−0.0177 (17)	−0.035 (2)

### Geometric parameters (Å, °)

**Table:**

O1—C12	1.358 (3)	C14—C15	1.383 (5)
O1—C16	1.416 (3)	C4—H4	0.9300
O2—C13	1.355 (3)	C5—H5	0.9300
O2—C17	1.436 (4)	C6—H6	0.9300
N1—C1	1.414 (3)	C7—H7A	0.9600
N1—C9	1.270 (3)	C7—H7B	0.9600
C1—C2	1.396 (3)	C7—H7C	0.9600
C1—C6	1.400 (4)	C8—H8A	0.9600
C2—C3	1.403 (3)	C8—H8B	0.9600
C2—C7	1.497 (3)	C8—H8C	0.9600
C3—C4	1.363 (4)	C9—H9	0.9300
C3—C8	1.504 (4)	C11—H11	0.9300
C4—C5	1.354 (5)	C14—H14	0.9300
C5—C6	1.400 (4)	C15—H15	0.9300
C9—C10	1.451 (4)	C16—H16A	0.9600
C10—C11	1.400 (3)	C16—H16B	0.9600
C10—C15	1.384 (4)	C16—H16C	0.9600
C11—C12	1.368 (3)	C17—H17A	0.9600
C12—C13	1.408 (3)	C17—H17B	0.9600
C13—C14	1.372 (3)	C17—H17C	0.9600
			
C12—O1—C16	118.63 (17)	C5—C6—H6	120.00
C13—O2—C17	118.4 (2)	C2—C7—H7A	109.00
C1—N1—C9	119.83 (19)	C2—C7—H7B	109.00
N1—C1—C2	118.28 (18)	C2—C7—H7C	109.00
N1—C1—C6	122.3 (2)	H7A—C7—H7B	109.00
C2—C1—C6	119.3 (2)	H7A—C7—H7C	109.00
C1—C2—C3	119.81 (18)	H7B—C7—H7C	109.00
C1—C2—C7	120.07 (16)	C3—C8—H8A	109.00
C3—C2—C7	120.11 (18)	C3—C8—H8B	109.00
C2—C3—C4	119.6 (2)	C3—C8—H8C	109.00
C2—C3—C8	121.02 (19)	H8A—C8—H8B	109.00
C4—C3—C8	119.4 (2)	H8A—C8—H8C	109.00
C3—C4—C5	121.7 (2)	H8B—C8—H8C	109.00
C4—C5—C6	120.3 (3)	N1—C9—H9	118.00
C1—C6—C5	119.3 (3)	C10—C9—H9	118.00
N1—C9—C10	123.5 (2)	C10—C11—H11	119.00
C9—C10—C11	121.3 (2)	C12—C11—H11	119.00
C9—C10—C15	120.1 (2)	C13—C14—H14	120.00
C11—C10—C15	118.5 (2)	C15—C14—H14	120.00
C10—C11—C12	121.1 (2)	C10—C15—H15	120.00
O1—C12—C11	125.7 (2)	C14—C15—H15	120.00
O1—C12—C13	114.90 (19)	O1—C16—H16A	109.00
C11—C12—C13	119.4 (2)	O1—C16—H16B	109.00
O2—C13—C12	114.7 (2)	O1—C16—H16C	109.00
O2—C13—C14	125.5 (3)	H16A—C16—H16B	109.00
C12—C13—C14	119.8 (3)	H16A—C16—H16C	109.00
C13—C14—C15	120.2 (3)	H16B—C16—H16C	109.00
C10—C15—C14	120.9 (2)	O2—C17—H17A	109.00
C3—C4—H4	119.00	O2—C17—H17B	109.00
C5—C4—H4	119.00	O2—C17—H17C	109.00
C4—C5—H5	120.00	H17A—C17—H17B	109.00
C6—C5—H5	120.00	H17A—C17—H17C	109.00
C1—C6—H6	120.00	H17B—C17—H17C	110.00
			
C16—O1—C12—C11	4.7 (3)	C8—C3—C4—C5	−180.0 (3)
C16—O1—C12—C13	−176.7 (2)	C3—C4—C5—C6	−0.6 (5)
C17—O2—C13—C12	−179.7 (2)	C4—C5—C6—C1	0.5 (4)
C17—O2—C13—C14	−0.3 (4)	N1—C9—C10—C11	8.8 (4)
C9—N1—C1—C2	−133.8 (2)	N1—C9—C10—C15	−166.8 (2)
C9—N1—C1—C6	49.3 (3)	C9—C10—C11—C12	−175.4 (2)
C1—N1—C9—C10	179.8 (2)	C15—C10—C11—C12	0.2 (3)
N1—C1—C2—C3	−178.66 (18)	C9—C10—C15—C14	174.7 (2)
N1—C1—C2—C7	2.3 (3)	C11—C10—C15—C14	−1.0 (4)
C6—C1—C2—C3	−1.7 (3)	C10—C11—C12—O1	179.8 (2)
C6—C1—C2—C7	179.2 (2)	C10—C11—C12—C13	1.2 (3)
N1—C1—C6—C5	177.5 (2)	O1—C12—C13—O2	−1.3 (3)
C2—C1—C6—C5	0.6 (4)	O1—C12—C13—C14	179.3 (2)
C1—C2—C3—C4	1.7 (3)	C11—C12—C13—O2	177.4 (2)
C1—C2—C3—C8	−178.9 (2)	C11—C12—C13—C14	−2.0 (3)
C7—C2—C3—C4	−179.3 (2)	O2—C13—C14—C15	−178.1 (2)
C7—C2—C3—C8	0.2 (3)	C12—C13—C14—C15	1.2 (4)
C2—C3—C4—C5	−0.5 (4)	C13—C14—C15—C10	0.3 (4)

### Hydrogen-bond geometry (Å, °)

**Table:**

*D*—H···*A*	*D*—H	H···*A*	*D*···*A*	*D*—H···*A*
C16—H16B···O2^i^	0.96	2.51	3.454 (3)	167

Symmetry codes: (i) −*x*+1, −*y*+3, −*z*.

## References

[bb1] Bruker (2005). *SADABS* Bruker AXS Inc., Madison, Wisconsin, USA.

[bb2] Bruker (2009). *APEX2* and *SAINT* Bruker AXS Inc., Madison, Wisconsin, USA.

[bb3] Farrugia, L. J. (1997). *J. Appl. Cryst.* **30**, 565.

[bb4] Farrugia, L. J. (1999). *J. Appl. Cryst.* **32**, 837–838.

[bb5] Sarfraz, M., Tariq, M. I. & Tahir, M. N. (2010). *Acta Cryst.* E**66**, o2055. 10.1107/S1600536810027832 PMC3007336 21588361

[bb6] Sheldrick, G. M. (2008). *Acta Cryst.* A**64**, 112–122. 10.1107/S0108767307043930 18156677

[bb7] Spek, A. L. (2009). *Acta Cryst.* D**65**, 148–155. 10.1107/S090744490804362X PMC2631630 19171970

[bb8] Tahir, M. N., Tariq, M. I., Ahmad, S., Sarfraz, M. & Ather, A. Q. (2010*a*). *Acta Cryst.* E**66**, o1562. 10.1107/S1600536810020933 PMC3007017 21587805

[bb9] Tahir, M. N., Tariq, M. I., Ahmad, S., Sarfraz, M. & Tariq, R. H. (2010*b*). *Acta Cryst.* E**66**, o2439. 10.1107/S1600536810033398 PMC3008100 21588761

[bb10] Tariq, M. I., Sarfraz, M., Tahir, M. N., Ahmad, S. & Hussain, I. (2010). *Acta Cryst.* E**66**, o2078. 10.1107/S1600536810028217 PMC3007434 21588378

